# Building a knowledge translation platform in Malawi to support evidence-informed health policy

**DOI:** 10.1186/s12961-015-0061-4

**Published:** 2015-12-08

**Authors:** Joshua Berman, Collins Mitambo, Beatrice Matanje-Mwagomba, Shiraz Khan, Chiyembekezo Kachimanga, Emily Wroe, Lonia Mwape, Joep J. van Oosterhout, Getrude Chindebvu, Vanessa van Schoor, Lisa M. Puchalski Ritchie, Ulysses Panisset, Damson Kathyola

**Affiliations:** Dignitas International, Zomba, Malawi; Ministry of Health, Department of Research, Public Health Institute of Malawi, Community Health Sciences Unit (CHSU), P.O. Box 65, Lilongwe, Malawi; University of North Carolina Project, Lilongwe, Malawi; Partners in Health, Neno, Malawi; Department of Nursing Sciences and Zambia Forum for Health Research, University of Zambia, School of Medicine, Lusaka, Zambia; Department of Medicine, University of Malawi College of Medicine, Blantyre, Malawi; Department of Medicine, University of Toronto, Toronto, Ontario Canada; University Health Network, Toronto, Ontario Canada; Li Ka Shing Knowledge Institute, St. Michael’s Hospital, Toronto, Ontario Canada; Faculdade de Medicina da Universidade Federal de Minas Gerais (School of Medicine-UFMG), Belo Horizonte, Brazil

**Keywords:** Evidence-informed health policy, Knowledge translation, Knowledge translation platform, Malawi, Research uptake

## Abstract

With the support of the World Health Organization’s Evidence-Informed Policy Network, knowledge translation platforms have been developed throughout Africa, the Americas, Eastern Europe, and Asia to further evidence-informed national health policy. In this commentary, we discuss the approaches, activities and early lessons learned from the development of a Knowledge Translation Platform in Malawi (KTPMalawi). Through ongoing leadership, as well as financial and administrative support, the Malawi Ministry of Health has strongly signalled its intention to utilize a knowledge translation platform methodology to support evidence-informed national health policy. A unique partnership between Dignitas International, a medical and research non-governmental organization, and the Malawi Ministry of Health, has established KTPMalawi to engage national-level policymakers, researchers and implementers in a coordinated approach to the generation and utilization of health-sector research. Utilizing a methodology developed and tested by knowledge translation platforms across Africa, a stakeholder mapping exercise and initial capacity building workshops were undertaken and a multidisciplinary Steering Committee was formed. This Steering Committee prioritized the development of two initial Communities of Practice to (1) improve data utilization in the pharmaceutical supply chain and (2) improve the screening and treatment of hypertension within HIV-infected populations. Each Community of Practice’s mandate is to gather and synthesize the best available global and local evidence and produce evidence briefs for policy that have been used as the primary input into structured deliberative dialogues. While a lack of sustained initial funding slowed its early development, KTPMalawi has greatly benefited from extensive technical support and mentorship by an existing network of global knowledge translation platforms. With the continued support of the Malawi Ministry of Health and the Evidence-Informed Policy Network, KTPMalawi can continue to build on its role in facilitating the use of evidence in the development and refinement of health policy in Malawi.

## Background

Policy and decision making in public health can be a difficult undertaking, as health policies developed and implemented by ministries of health affect large populations. Low-income countries such as Malawi have scarce resources to address their health system challenges and need high-quality evidence to use available resources efficiently. If health sector managers and policymakers overlook evidence on the root causes of problems or effective potential solutions to address these problems in different contexts, they risk wasting precious resources on inadequately designed programmes and policies.

Beginning with the 2004 Ministerial Summit in Mexico [1] and, more recently, the Global Symposium on Health Systems Research Beijing [2] and Cape Town Statements [3], there have been numerous calls to improve the use of evidence in health policymaking. In 2005, the WHO created the Evidence-Informed Policy Network (EVIPNet), which promotes the systematic use of health research evidence in policymaking in low- and middle-income countries [4]. Under EVIPNet’s inspiration and encouragement, national knowledge translation platforms have been developed throughout Africa, the Americas, Eastern Europe, and Asia. Knowledge translation platforms bring together policymakers, researchers, implementers, civil society groups, and other key health system stakeholders to facilitate the utilization of the best available local and international evidence to improve policy, its implementation and in refining national health research agendas. Building on important historical case studies of well-established knowledge translation platforms in Zambia [5], Uganda [6], and Cameroon [6], this commentary discusses the approaches, activities and early lessons learned from the development of a knowledge translation platform in Malawi (KTPMalawi).

## Development of KTPMalawi

In 2012, the Malawi Ministry of Health’s Head of Research and a small multidisciplinary group began discussing how to enhance the use of evidence in national health policy. Evidence-informed health policymaking is an approach to policy decisions that aims to ensure that decision making is well-informed by the best available research evidence [7]. These initial meetings led to a partnership between the Malawi Ministry of Health and Dignitas International, a medical and research non-governmental organization with a long-term presence in Malawi. Together, members of the Malawi Ministry of Health and Dignitas International have spearheaded the development of KTPMalawi.

Utilizing the lessons learned from regional knowledge translation platforms [5], an initial rapid stakeholder mapping exercise was undertaken to understand how various institutions, from civil society to government ministries, use, demand and absorb research, the nature of researcher-policymaker interactions, and the kinds of opportunities that exist for linking research and policy processes. This exercise led to two capacity building workshops, which utilized the Supporting the Use of Research Evidence (SURE) Guides and the Supporting Policy Relevant Reviews and Trials tools [8, 9]. A workshop for policymakers focused on building capacity to interpret, assess and utilize research evidence. Researchers assisted in this workshop’s facilitation. A subsequent workshop for researchers built capacity in developing research syntheses such as policy briefs and research summaries. Policymakers led the facilitation of this workshop. These two initial workshops helped to galvanize support for KTPMalawi and created an opportunity for stakeholders to be engaged in the planning of subsequent activities.

In June 2012, KTPMalawi was presented to, and endorsed by, the full senior management team of the Malawi Ministry of Health. KTPMalawi then convened its inaugural Steering Committee meeting. Comprised of Ministry of Health policymakers, implementers, multi-disciplinary researchers from academic institutions and health sector organizations, and civil society members, this Steering Committee planned KTPMalawi objectives and activities and considered several distinctive KTPMalawi structural models. A short structured prioritization process was developed and utilized asking Steering Committee members to rank possible Community of Practice content areas using themes such as (1) policymaker demand for evidence, (2) the availability of new or emerging evidence in the content areas, and (3) content access and expertise in Malawi. This ranking exercise assisted the Steering Committee in the prioritization of two Communities of Practice in (1) pharmaceutical supply chain management and (2) the integrated management of non-communicable and communicable diseases. The Steering Committee intentionally defined the community of practices broadly to allow members, experts in each of these two areas, to narrow the content scope of each Community of Practice.

Two Communities of Practice meetings were then convened, bringing researchers, policymakers and implementers who work in these two content areas together. Again, utilizing facilitation guides and templates developed by the SURE program [8], each Community of Practice discussed potential policy problems that could be tackled by the development of an evidence brief for policy, a research synthesis in a user-friendly format, offering evidence-informed policy options [10]. The two Communities of Practice narrowed their content scope for the first two evidence briefs for policy to (1) improving data quality and utilization in the pharmaceutical supply chain and (2) improving the screening and treatment of hypertension within HIV-infected populations. Several Community of Practice members were selected to lead the authorship of each evidence brief for policy with authors coming from research, practice and policy communities.

A weeklong training was then held for both author groups. This training was facilitated by Canadian, Zambian and Malawian knowledge translation experts and focused on:

1. Understanding factors that impact evidence quality.
2. Locating and assessing evidence, using GRADE [11], AMSTAR [12], and AGREE II [13].
3. Conducting and utilizing systematic reviews.
4. Developing evidence briefs for policy [8].

Subsequently, the development of two evidence briefs for policy began, utilizing the model previously developed by the SURE program and EVIPNet [8]. The evidence brief, aimed at improving the screening and treatment of hypertension within HIV-infected populations, was completed and used as the primary input into a structured deliberative dialogue. This dialogue brought together 52 HIV and hypertension policymakers, researchers, practitioners, and civil society members for discussion on the magnitude of undiagnosed hypertension within Malawi’s HIV-infected population, factors that underlie the problem, potential policy options to address the problem, and implementation considerations that would need to be considered should one of the proposed policy options be taken forward. Attendees of the deliberative dialogue concluded that more local evidence was needed and noted that all of the four potential policy options are currently being piloted within Malawi. It was agreed during the dialogue that the organizations that are piloting these policy options would look to align indicators across the pilots to maximize comparability of the options during subsequent follow-up discussions. These dialogues have been shown to increase the likelihood that research evidence will be used in policymaking [14–16].

## Lessons learned

There are several lessons to highlight from initial KTPMalawi activities.

The development of KTPMalawi under the guidance of the Ministry of Health’s Head of Research reinforces the need for strong leadership and decision-making authority within knowledge translation platforms. This has been emphasized by other platforms as an enabling factor [5, 17]. This leadership supports bridging between the research and policy communities and supports the effective integration of the platform into existing national policy structures. The Head of Research advocated for the hiring of a full time Knowledge Translation Coordinator within the Malawi Ministry of Health. This position was created through core Ministry funds and filled in early 2014. Cultivating key champions for evidence-informed health policy could be a foundational step in the formation of new knowledge translation platforms.

The development of KTPMalawi has benefited greatly from the capacity and lessons learned of previously established knowledge translation platforms. A member of the Zambian Knowledge Translation Platform, the Zambia Forum for Health Research, attended KTPMalawi’s inaugural Steering Committee meeting, facilitated the author training and shared lessons learned as well as technical expertise. In addition, KTPMalawi’s Knowledge Translation Coordinator travelled to Uganda to receive coaching and mentorship from Uganda’s Knowledge Translation Platform, the Regional East African Community Health Policy Initiative – Uganda [18]. KTPMalawi has also been brought into the existing EVIPNet global community, attending workshops, including the International Forum on Evidence Informed Health Policymaking [19], which built knowledge translation capacity and further strengthened linkages with regional and global knowledge translation platforms. Future knowledge translation platform development should continue to leverage international capacity, especially during the early stages.

Several papers have discussed the potential benefits and drawbacks of differing knowledge translation platform structural models [5, 6, 17]. KTPMalawi was formed out of a unique partnership between the Malawi Ministry of Health and Dignitas International, a medical and research non-governmental organization. During KTPMalawi’s initial Steering Committee meeting a decision was taken to house KTPMalawi within the Ministry of Health’s research department. The partnership with Dignitas exists through the implementation of KTPMalawi’s activities and has not been formalized. As this initiative matures and further outputs are developed this partnership will be reviewed and formalized as necessary. Several benefits have been noted from this partnership. With KTPMalawi housed within the Malawi Ministry of Health, the initiative has benefited from being viewed as a Government initiative. This has opened doors for in-country funding, allowed key policymakers to take part in workshops and in the development of evidence briefs for policy and increased the overall support for this initiative. Dignitas International’s involvement has facilitated external start-up funding and ongoing technical assistance. Dignitas International’s involvement has also facilitated interactions with the Malawi and international research community, through the development of conference presentations and research proposals that support KTPMalawi’s knowledge translation role. As KTPMalawi matures and produces additional outputs, its Steering Committee will further discuss how best to incorporate this initiative into Malawi’s health research system.

As has been discussed elsewhere, building knowledge translation capacity amongst platform stakeholders is critical to long-term success [5, 6, 17, 20]. As previously described, KTPMalawi has undertaken a series of activities to build the capacity of its stakeholders and in particular the evidence brief authors. KTPMalawi has specifically focused on building the capacity of early stage researchers, implementers and policymakers who have long-term interest in knowledge translation and evidence-informed health policy in Malawi. Because evidence brief authors are taking on this writing role in addition to their regular job responsibilities, we found that bringing the authors together, away from their usual working place, contributed to evidence brief progress.

KTPMalawi’s activities have been funded by small grants from the International Development Research Centre, the WHO, the Malawi National Commission for Science and Technology and USAID-Malawi. All funds have been channelled through Dignitas to support KTPMalawi activities. A lack of sustained initial funding, however, slowed the early development of KTPMalawi. KTPMalawi activities paused several times as relatively small amounts of funding were sought through international and local funding agencies. This lack of funding has been cited by other knowledge translation platforms as an obstacle to sustainability [17]. These pauses temporarily interrupted KTPMalawi’s momentum, making subsequent activities more difficult to organize and undertake. Inasmuch as is possible, future knowledge translation platforms may benefit from delaying initial activities until sustained initial funding over the course of several years can be secured. Once a knowledge translation platform exists, funding opportunities from both research initiatives and intervention projects should be explored. We have noted that researchers in Malawi are increasingly valuing knowledge translation and are including dedicated budget lines in new proposals.

As this initiative continues, KTPMalawi will need to utilize implementation research methodologies and strengthen its use of previously developed knowledge translation platform monitoring and evaluation methods to capture outcomes and impacts on the policy and research environments, in a continuous effort to review, adjust and improve health policies in Malawi [17, 21].

## Conclusions

With the support of EVIPNet and regional knowledge translation platforms, KTPMalawi has been shown to be a viable model for supporting evidence-informed national health policy, with a strong partnership between policymakers and researchers. Lessons learned from the original two communities of practice, some content specific and others more process oriented and therefore generalizable, will facilitate improved efficiency of future communities of practice moving forward. With adequate funding, KTPMalawi will be able to explore processes to better include civil society through participation in structured dialogues and with the help of public media. While still early in its development, KTPMalawi has benefitted from strong Ministry of Health support and has built knowledge translation capacity within key stakeholders groups.

## Abbreviations

KTPMalawi: Knowledge translation platform in Malawi
EVIPNet: Evidence-informed policy network
SURE: Supporting the use of research evidence
